# Influence of Egr-1 in Cardiac Tissue-Derived Mesenchymal Stem Cells in Response to Glucose Variations

**DOI:** 10.1155/2014/254793

**Published:** 2014-05-22

**Authors:** Daniela Bastianelli, Camilla Siciliano, Rosa Puca, Andrea Coccia, Colin Murdoch, Antonella Bordin, Giorgio Mangino, Giulio Pompilio, Antonella Calogero, Elena De Falco

**Affiliations:** ^1^Department of Medical-Surgical Sciences and Biotechnologies, Faculty of Pharmacy and Medicine, University “Sapienza”, Corso della Repubblica 79, 04100 Latina, Italy; ^2^Center for Life Nano Science@Sapienza, Istituto Italiano di Tecnologia, Viale Regina Elena 291, 00161 Rome, Italy; ^3^School of Medicine, Aston University, Aston Triangle, Birmingham B4 7ET, UK; ^4^Laboratory of Vascular Biology and Regenerative Medicine, Centro Cardiologico Monzino, IRCCS, Via C. Parea 4, 20138 Milan, Italy; ^5^Department of Clinical Sciences and Community Health, Università degli Studi di Milano, Via Francesco Sforza 35, 20122 Milan, Italy

## Abstract

Mesenchymal stem cells (MSCs) represent a promising cell population for cell therapy and regenerative medicine applications. However, how variations in glucose are perceived by MSC pool is still unclear. Since, glucose metabolism is cell type and tissue dependent, this must be considered when MSCs are derived from alternative sources such as the heart. The zinc finger transcription factor Egr-1 is an important early response gene, likely to play a key role in the glucose-induced response. Our aim was to investigate how short-term changes in *in vitro* glucose concentrations affect multipotent cardiac tissue-derived MSCs (cMSCs) in a mouse model of Egr-1 KO (Egr-1^−/−^). Results showed that loss of Egr-1 does not significantly influence cMSC proliferation. In contrast, responses to glucose variations were observed in wt but not in Egr-1^−/−^ cMSCs by clonogenic assay. Phenotype analysis by RT-PCR showed that cMSCs Egr-1^−/−^ lost the ability to regulate the glucose transporters GLUT-1 and GLUT-4 and, as expected, the Egr-1 target genes VEGF, TGF**β**-1, and p300. Acetylated protein levels of H3 histone were impaired in Egr-1^−/−^ compared to wt cMSCs. We propose that Egr-1 acts as immediate glucose biological sensor in cMSCs after a short period of stimuli, likely inducing epigenetic modifications.

## 1. Introduction

Mesenchymal stem cells (MSCs) are widely distributed in the body and are an important source of tissue formation and regeneration. Cardiac tissue-derived MSCs (cMSCs) are resident in the heart, within the cardiac stromal cell compartment, a key component of the stem cell niche [1, 2].

Glucose is the main fuel source for MSCs, whose pool and metabolism are influenced in both physiological and pathological conditions [3, 4].

The way in which glucose variations in MSCs exert their effects on stem cell pool is a highly interesting issue. Elevated concentrations of glucose have been demonstrated to impair original important cellular functions, such as apoptosis, cell viability, and proliferation as well as colony forming ability both* in vitro* and* in vivo* of MSCs regardless of their tissue of origin [3, 5, 6]. In contrast, glucose reduction improves all the aforementioned properties, due to the overall beneficial effects of caloric restriction reported in MSCs such as a decrease in cell death, senescence, and aging [7].

Glucose depletion seems also able to augment the* in vivo* ability of MSCs to repair infarct myocardium [7]. In addition, in rat multipotent adult progenitor cells but not in human MSCs hyperglycemia has been correlated to the suppression of specific growth factors [8, 9], thus highlighting the fact that differences in the effect of glucose on MSCs might exist between species. Moreover, hyperglycemia has been demonstrated to alter gene expression and even to drive the mesodermal transdifferentiation of MSCs towards a preferential adipogenic pathway at the expense of the chondrogenic and osteogenic lineages [6, 10]. In other cell systems such as endothelial cells, transient hyperglycemia has been reported to cause relevant transcriptional changes, thus offering an explanation for the persistent presence of systemic complications in diabetic patients returning to normal glycemic levels [11].

Taken together these observations have suggested that damage caused by a dysregulation in the glucose level tolerance may affect stem cell properties in a long term and irreversible fashion.

The zinc finger transcription factor Egr-1 is a member of a group of early response genes [12, 13]. A variety of stimuli, ranging from hormones to UV light, are able to activate Egr-1, affecting several cellular processes such as proliferation, differentiation, apoptosis, and growth [14–17]. Egr-1 is also responsive to glucose [18]. Glucose induces early growth response gene (Egr-1) expression in pancreatic beta cells, [19], a stress signaling for all cells. The involvement of Egr-1 in insulin production of *β*-pancreatic cells [12], thus mediating the insulin resistance process in mice, has been reported [20]. Recently, human bone marrow-derived MSCs genetically modified to express insulin using the glucose-responsive Egr-1 promoter have been tested for diabetes therapy [21].

Egr-1 is highly expressed in MSCs, representing a convergence point of multiple signaling pathways, mainly involved in the production of growth factors critical to their regenerative properties [22].

To the best of our knowledge, nothing has been reported so far concerning the role of Egr-1 in response to glucose stress in cMSCs. Thus, the question of whether Egr-1 has any role in the biological response to glucose in cMSCs still remains unexplored. In the present report, we have investigated the* in vitro* short-term effects of the response of Egr-1 to glucose in cMSCs.

## 2. Materials and Methods

### 2.1. Animal Model and Surgery

The study was conducted using C57BL/6 wild-type (wt) and Egr-1 deficient mice (Egr-1^−/−^) obtained as previously reported [13]. Mice were handled and sacrificed in compliance with the European Convention on Animal Care. They have also received human care in accordance with the principles of Laboratory Animal Care and the Guide for the Care and Use of Laboratory Animals (ILAR 1996). The study protocol was approved by the Local Ethical Committee.

### 2.2. Cardiac Tissue-Derived Mesenchymal Stem Cell (cMSCs) Isolation and Culture

Cardiac tissue-derived mesenchymal stem cells (cMSCs) were isolated from hearts of 2-month-old wt and Egr-1^−/−^ homozygous mice. Hearts were excised and kept in a sterile Falcon tube with complete biopsy medium (Iscove's modified Dulbecco's medium (IMDM) supplemented with 20% fetal bovine serum (FBS), 100 U/mL penicillin, and 100 *μ*g/mL streptomycin) and transported to the laboratory at 4°C and processed within 4 h.

Specimens were then washed with PBS supplemented with 5% amphotericin-B and 5% penicillin-streptomycin and minced using a sterile scalpel in a 100 mm petri dish into small pieces, and the fragments were transferred into a clean tube filled with the enzymatic digestion solution containing 1 : 1 Ham's-F12, 0.05% Trypsin, 0.02% EDTA, and 3 mg/mL collagenase type-I. Samples were incubated at 37°C in a shaking water bath for 2-3 hours [23]. The enzymatic digestion was blocked with PBS/20%FBS and the digested solution was filtered with a 70 *μ*m cell strainer. Cell suspension was centrifuged at 456 ×g for 10 min at room temperature. Cellular pellet was transferred in a sterile tube and resuspended in complete biopsy medium and seeded in a T25 ventilated flask and incubated at 37°C with 5% CO_2_. After 24 h, half of the medium was removed and replaced with fresh complete medium. When cells reached the 70–80% confluence, they were detached by 0.05% Trypsin and 0.02% EDTA, centrifuged at 377 ×g for 5 min, and counted by Trypan Blue. Cells were seeded in complete growth medium (IMDM supplemented with 10% FBS, 1% penicillin-streptomycin, 1% L-glutamine, 1% nonessential amino acids, and 1% amphotericin-B) at a density of 6.000 cells/cm^2^. Half of the medium was changed every 3 days. Experiments were performed expanding cMSCs between passages 3–5.

### 2.3. FACS Analysis

For FACS analysis, cMSCs were treated as we previously described with little modifications [23]. Briefly, cells were stained for 30 min with the following conjugated primary antibodies: PE anti-mouse CD-31 (clone 390, Cat. number 102408 Biolegend), APC anti-mouse CD-45 (clone 30-F11, Cat. number 103112 Biolegend), and FITC anti-mouse Ly-6A/E (Sca-1) (clone D7, Cat. number 108106 Biolegend). Thereafter, cells were washed with cold PBS-FACS flow and acquired using FACSAria II (B&D, San Jose, CA, USA). For detection of apoptosis, cMSCs from wt and Egr-1^−/−^ were cultured in basal medium for 24 and 48 hours and then collected and stained for 30 minutes with Annexin V-FITC antibody (Becton and Dickinson Cat. number 556420) and counterstained with propidium iodide (10 ng/mL, Sigma) in order to exclude dead cells. Identical instrument settings were used for all conditions. All cytofluorimetric data were acquired by using DiVa Software (v6.1.1, B&D, San Jose, CA, USA) and analyzed by Flowing Software (v2.5.1, Turku Centre for Biotechnology, Turku, Finland).

### 2.4. cMSC Conditioning with Glucose

cMSCs were detached by 0.05% Trypsin/0.02% EDTA, counted by Trypan blue, seeded in complete growth medium, and allowed to adhere overnight. Thereafter, cells were starved with IMDM supplemented with 0.2% FBS and after 12 h the medium was changed and replaced with fresh Dulbecco's modified Eagle medium (DMEM) containing the following glucose concentrations: (I) no glucose (DMEM no glucose, Gibco Cat. number 11966-025); (II) 25 mM (DMEM 25 mM glucose, Sigma Cat. number D5671); (III) 50 mM (DMEM 25 mM glucose with a further supplement of glucose to reach 50 mM).

### 2.5. Proliferation Assay

cMSC proliferation rate was evaluated by Trypan Blue exclusion assay [24]. Briefly, cMSCs were seeded in quintuplicate at density of 2500 cells/cm^2^ in 24-well plate and subjected to different concentrations of glucose as described above for 0, 48, and 168 h (7 days).

### 2.6. Colony Forming Unit Fibroblasts Assay (CFU-F)

For clonogenic assay, cMSCs were seeded at low density (40 cells/cm^2^) in a 60 mm petri dish and subjected to different concentrations of glucose as described before. After 2 weeks of incubation, the petri dish was gently washed with PBS, fixed with 4% paraformaldehyde, and incubated for 2 min with absolute Giemsa (Sigma, St. Louis, MO, USA) and then washed again in distilled water and incubated for 13 min with diluted Giemsa (1 : 20 distilled water, Sigma, St. Louis, MO, USA) [23]. The petri was washed with distilled water and cell clusters with a diameter ≥5 mm were considered colonies observed and counted by optical microscope.

### 2.7. Gene and Protein Expression Profiling

RNA was extracted from preconditioned cMSCs. Briefly, cells were plated at density of 6.000 cells/cm^2^ and treated as described before. After 2 h, they were detached with a scraper and RNA was extracted using* RNeasy MicroKit* (Quiagen, Hilden, Germania) [24–27]. cDNA synthesis was performed from 1 *μ*g RNA by using* High Capacity cDNA Reverse Transcription Kit*. Real-time PCR was carried out by using SensiMix SYBR Hi-ROX kit (Bioline, London, UK). The relative ratio and standard deviation between treated samples were calculated using comparative *C*_*t*_ method (ΔΔ*C*_*t*_ value). Transcript levels were assessed by qPCR using Sensimix Sybr Green in a MiniOpticon instrument equipped with CFX software (Biorad) for 40 thermal cycles (95°C for 15 seconds, 60°C for 10 seconds, and 72°C for 30 seconds). The set of gene analyzed and the primers sequence are listed in Table 1. 18S was used as housekeeping gene and the expression levels of cells in standard culture conditions (25 mM glucose) as the reference for normalization.

### 2.8. Western Blot

After glucose conditioning in culture, cMSCs were washed. For nuclear extracts, cells were trypsinized, rinsed with PBS, and collected by centrifugation. Cells were then suspended in hypotonic buffer (10 mM HEPES, pH 7.9, 10 mM KCl, 0.1 mM EDTA, and 0.1 mM EGTA) and placed on ice for 15 min. NP40 was added to a final concentration of 0.5%. Cells were spun top speed for 30 s before the supernatant (cytoplasmic fraction) was collected. The remaining pellet was washed with hypotonic buffer, resuspended in ice-cold RIPA buffer (10 mM Tris pH 8, 1% Triton, 0.1% SDS, 0.1% Deoxycholate, 140 mM NaCl, and 1 mM EDTA) and a cocktail of protease inhibitors (1 mM DTT, 1 mM PMSF, and 1 Protease inhibitor tablet/10 mL Sigma), placed on ice for 20 min, and sonicated and spun at 15000 ×g for 15 min to remove debris and collect the supernatant (nuclear fraction). Supernatants were boiled for 5 min in a Laemmli sample buffer. Equivalent protein quantities, as determined by Bradford, were loaded on each gel. Proteins were separated by 15% SDS-PAGE electrophoresis and electrotransferred to polyvinylidene difluoride membranes and incubated o/n at 4°C with the primary antibodies against rabbit acetyl histone H3 (1 : 1000, Upstate Cat. N. 06-599) and mouse histone H3 (1 : 5000, Upstate Cat. number 05-499). A horseradish peroxidase-conjugate secondary antibody is incubated for 1 h at RT (Amersham mouse Cat. number NA93IVS, rabbit NA934VS). The membranes were developed by enhanced chemiluminescence kit (ECL, Amersham). Densitometric analysis has been performed with ImageJ densitometry software.

### 2.9. Statistical Analysis

Statistical analysis was performed and the data plotted using GraphPad Prism 5 software (San Diego, USA). The independent sample two-tailed *t*-test with associated 95% confidence intervals was used to compare the single data. For multiple comparisons, the one analysis of variance (ANOVA) test and the Bonferroni post hoc test were used. *P* values <0.05 were considered to be significant. Experiments were performed three times. Data are expressed as means ± SEM unless specified.

## 3. Results

### 3.1. Proliferation Responses to Glucose Administrations

cMSCs derived from wt and Egr-1^−/−^ mice were firstly isolated and phenotypically analyzed by cytofluorimetry according to the expression of differentiation antigens on the surface of murine cardiac stromal cells as previously reported [28].

**Figure 1:**
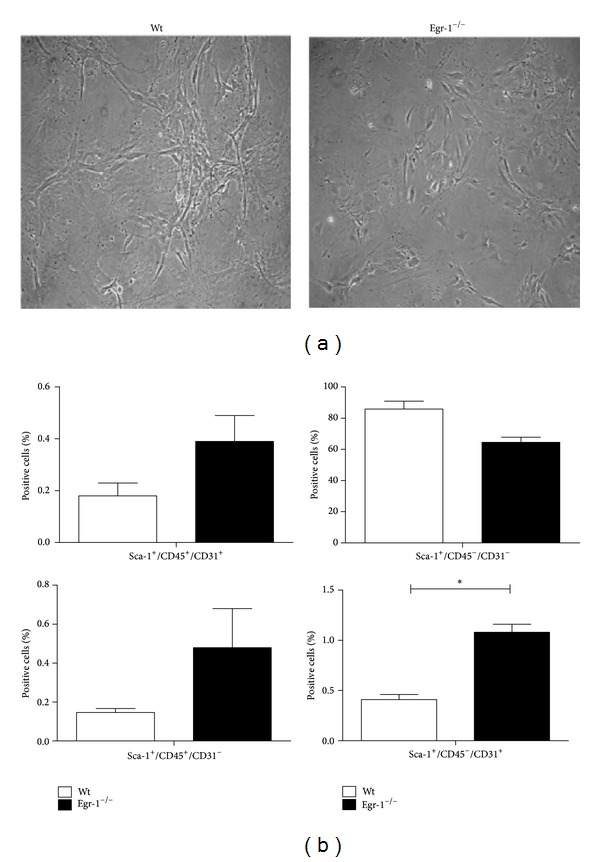
(a) Optical images of cMSCs derived from wt and Egr-1^−/−^ mice showing no difference in cell morphology. Magnification 10x. (b) Immunophenotype of wt and Egr-1^−/−^ cMSCs. FACS analysis histograms display that, among the four subsets, only the percentage of Sca-1^+^/CD45^−^/CD31^+^ cells was higher in Egr-1^−/−^ than in wt cMSCs. *P < 0.05. The error bars represent the SD.

As displayed in Figure 1(a), no difference in cell morphology could be found between cMSCs derived from wt or Egr-1^−/−^ mice when observed under inverted-phase microscope. In addition, after isolation only the percentage of the Sca-1^+^/CD45^−^/CD31^+^ subset was statistically significant higher in Egr-1^−/−^ cMSCs compared to wt (*P* = 0.02, Figure 1(b)). A similar phenotype with regard to the Sca-1^+^/CD45^−^/CD31^−^, the Sca-1^+^/CD45^+^/CD31^+^, and the Sca-1^+^/CD45^+^/CD31^−^ subset was observed in wt and Egr-1^−/−^ cMSC.

**Figure 2:**
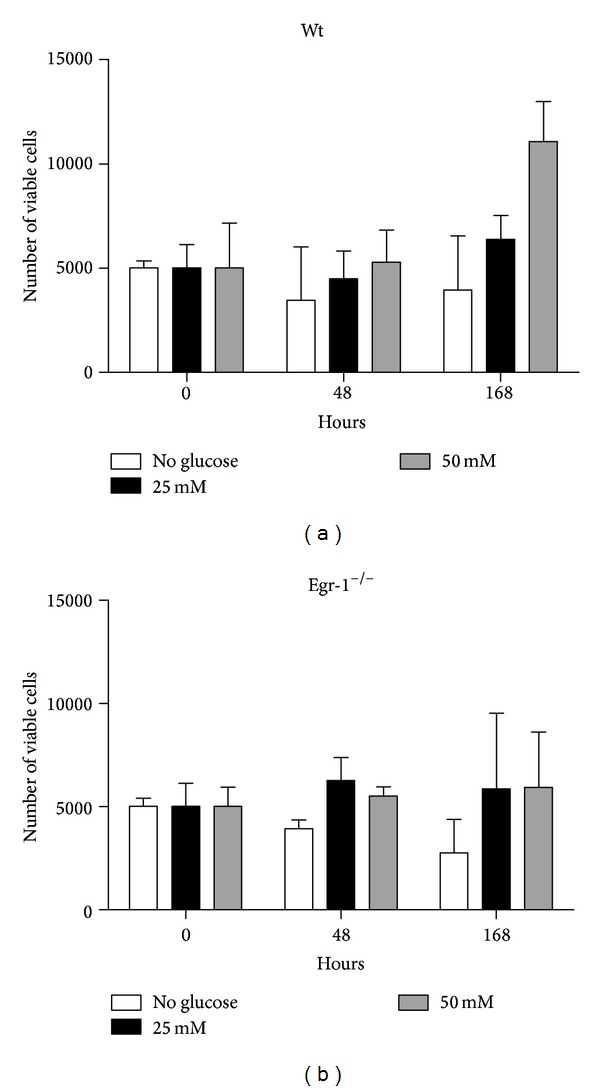
Cell proliferation of cMSCs subjected to glucose variations in culture and in presence of 0.2% FBS and measured by Trypan Blue exclusion assay by counting viable cells. No significant difference in cell proliferation can be observed either at increased concentrations or time exposure of glucose and between wt and Egr-1^−/−^ cMSCs. The error bars represent the SD.

Afterwards, cMSCs were administered with different concentrations of glucose* in vitro*. Cell proliferation was then assessed by Trypan Blue exclusion assay at different time intervals (0, 48 and 168 hours). Experiments were performed in the presence of low serum (0.2% FBS), in order to avoid possible interference of the serum on cell proliferation, thus masking the effect of glucose. None of the different glucose concentrations at the different exposure times caused a statistically significant difference (*P* > 0.05) in both wt and Egr-1^−/−^ cMSCs (Figure 2). Interestingly, cMSCs survived in absence of glucose up to 7 days as shown in Figure 2. As control, growth at basal conditions (25 mM glucose and 10% FBS) of Egr-1^−/−^ cMSCs was significantly enhanced compared to wt cMSCs up to 48 hours (Supplementary Figure 2, available online at http://dx.doi.org/10.1155/2014/254793). Nevertheless, the decrease in cell growth at basal conditions in wt cMSCs cannot be imputable to an increase in cell apoptosis as shown in Supplementary Figure 2.1, where the percentage of Annexin-V^+^ cMSCs (early apoptosis) is comparable in wt and Egr-1^−/−^ mice at both 24 and 48 hours (*P* = 0.33 at 24 hours; *P* = 0.37 at 48 hours). In addition, propidium iodide counterstaining, used to exclude late apoptosis and necrotic cells, shows no statistically significant difference between cMSCs derived from wt and Egr-1^−/−^ mice (*P* = 0.24 at 24 hours and *P* = 0.76 at 48 hours).

### 3.2. Colony Forming Ability after Glucose Administration

**Figure 3:**
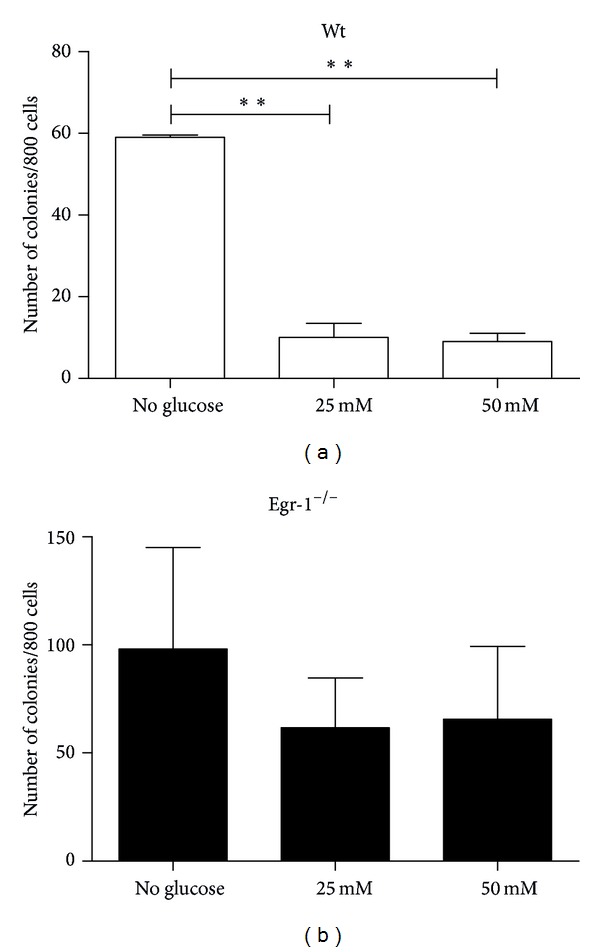
The number of clones significantly decreases in wt cMSCs at increased concentrations of glucose. Differently, glucose variations does not influence the clonogenic capacity of cMSCs in Egr-1^−/−^ cMSCs. **P < 0.01.

We investigated whether glucose variations could influence the clonogenic capacity of cMSCs in presence or absence of the Egr-1 gene. Under low serum concentration, only cMSCs derived from Egr-1^−/−^ were able to generate clones (data not shown). This suggests that wt cMSCs require a higher concentration of serum for their clonogenic ability. In fact, increased concentrations of glucose in culture significantly decrease the number of colonies formed by wt cMSCs compared to the absence of glucose (25 mM and 50 mM all *P* < 0.01, Figure 3). Glucose variations did not affect the ability of forming colonies in Egr-1^−/−^ cMSCs (all *P* > 0.05).

### 3.3. Transcriptional Activity of Egr-1^−/−^ and Egr-1^−/−^ Target Genes

We next aimed to evaluate whether in cMSCs derived from wt and Egr-1^−/−^ mice the glycolytic stimuli could be reflected into variations of Egr-1 mRNA levels and its transcriptional coactivator with histone acetyl transferase activity, p300 [11, 29, 30]. We also investigated the canonical GLUT-1 and the cardiac specific GLUT-4 [31] glucose transporters and the specific glucose and Egr-1 responsive growth factor genes VEGF and TGF*β*-1.

To this purpose, cells were administered with different concentrations of glucose (no glucose, 25, and 50 mM) for 2 hours and the gene expression profile was analyzed by RT-PCR. The 25 mM dose culture condition, which is the standard concentration of glucose employed for basal growth of cMSCs in our culture conditions, was set as the reference dose for normalization of results.

**Figure 4:**
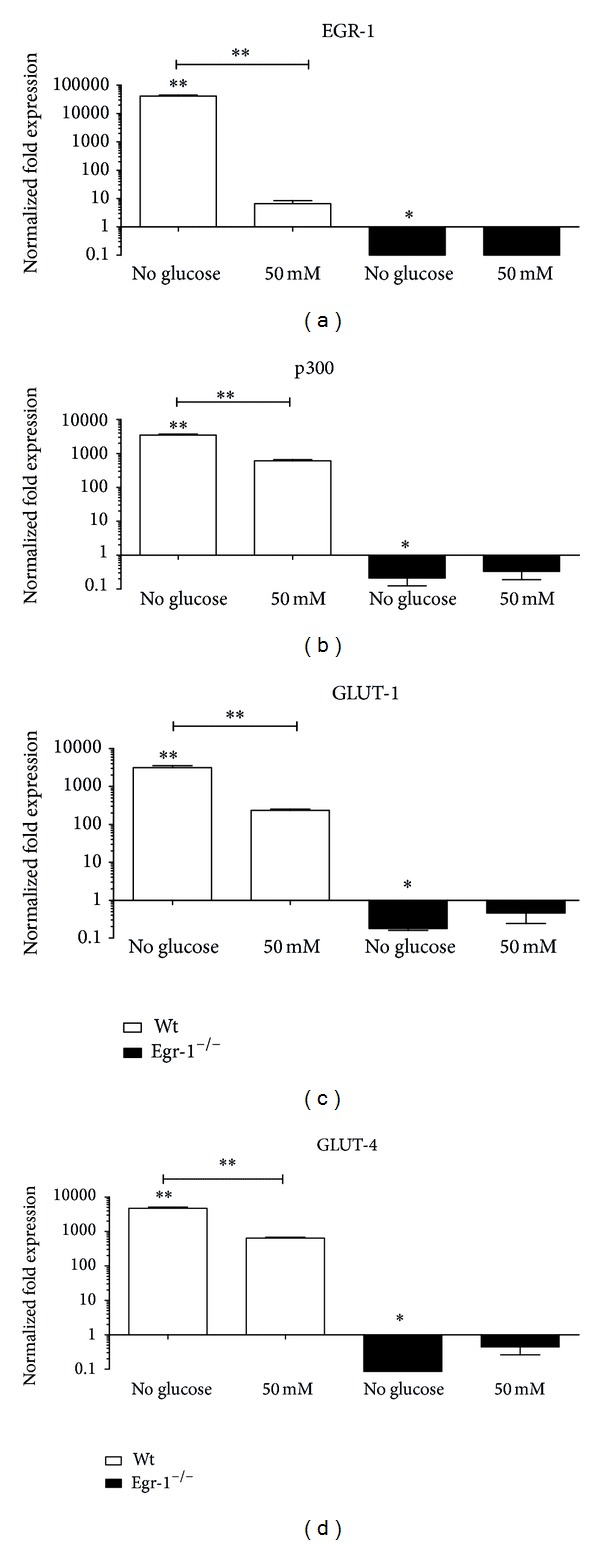
Gene expression profile of cMSCs after* in vitro* glucose conditioning. (a) Egr-1 mRNA levels in wt cMSCs are significantly increased in absence of glucose and compared to the 50 mM condition. A similar fashion can be observed as well with regard to (b) p300, (c) GLUT-1, and (d) GLUT-4. Differently, the same mRNA gene levels are significantly downregulated or unaffected in Egr-1^−/−^ cMSCs compared to the basal condition. The 25 mM condition was considered as reference. *P < 0.05. **P < 0.01.

A striking upregulation of Egr-1 transcription (*P* < 0.01) was induced in absence of glucose, which was significantly higher than that obtained after 2 hours of 50 mM conditioning in wt cMSCs (Figure 4(a)). Egr-1 mRNA level was absent in Egr-1^−/−^ cMSCs in all conditions, as expected.

Similarly (Figure 4(b)), a significant upregulation of p300 (*P* < 0.01), GLUT-1 (*P* < 0.01), and GLUT-4 (*P* < 0.01) transcriptional levels was observed in wt cMSCs in absence of glucose and to a lesser extent after 50 mM of glucose (all *P* < 0.01).

Interestingly, statistically significant variations of p300, GLUT-1, and GLUT-4 mRNA levels were observed also in Egr-1^−/−^ cMSCs in absence of glucose (*P* < 0.01) compared to the basal level, but not after the 50 mM glucose dosage.

**Figure 5:**
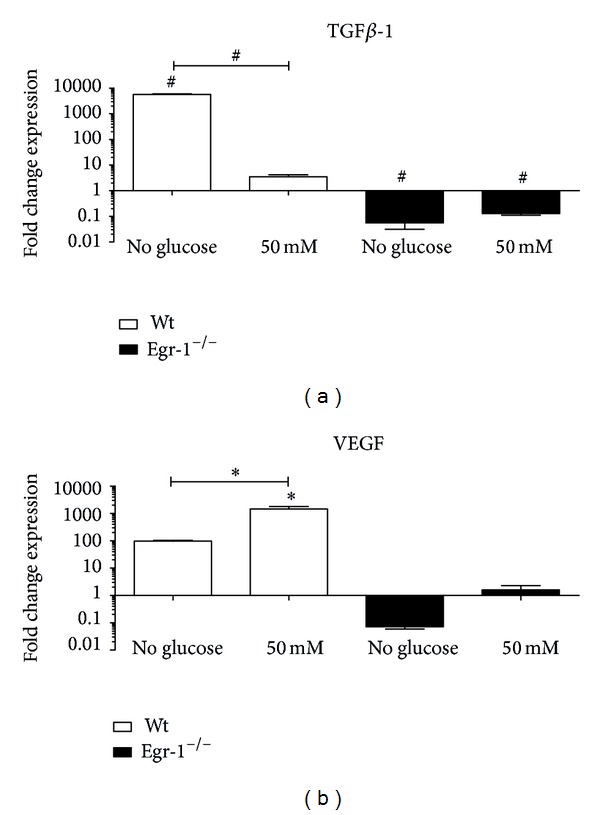
(a) The absence of glucose in wt cMSCs is able to induce a striking upregulation of TGF*β*-1, whose mRNA levels significantly decrease at 50 mM glucose. In Egr-1^−/−^ cMSCs mRNA levels are significantly downregulated compared to the basal condition both in absence and at 50 mM of glucose. (b) VEGF mRNA levels are significantly increased at the highest concentration of glucose in wt cMSCs, which is also statistically significantly increased compared to the absence of glucose, whereas in absence of Egr-1 gene this fluctuation is abolished. The 25 mM condition was considered as reference. *P < 0.05. ^#^ *P* < 0.0001.

In addition (Figure 5(a)), glucose deprivation of wt cMSCs strongly upregulated the TGF*β*-1 mRNA levels (*P* < 0.001), which significantly decreased at the glucose concentrations of 50 mM (*P* < 0.001). Conversely, a significant decrease in TGF*β*-1 mRNA levels was detected in absence (*P* < 0.001) and at 50 mM of glucose (*P* < 0.001) in Egr-1^−/−^ cMSCs.

However, in wt cMSCs the conditioning with 50 mM of glucose but not its absence induced upregulation of VEGF mRNA levels (*P* < 0.05). In Egr-1^−/−^ cMSCs, the VEGF mRNA levels were not significantly deregulated (Figure 5(b)).

### 3.4. Epigenetic Modifications

Finally, given that glucose has been demonstrated to induce epigenetic modifications [11] and that p300, the Egr-1 transcriptional coactivator with histone acetyl transferase activity, is downregulated in Egr-1^−/−^ cMSCs, we have asked whether this could be the result of epigenetic changes.

**Figure 6:**
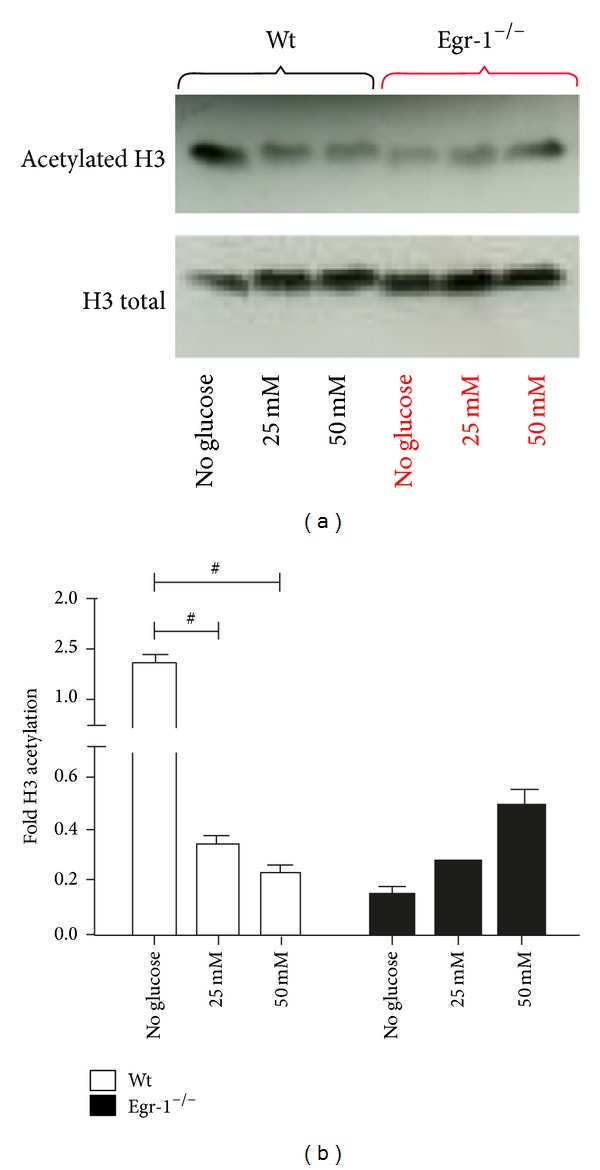
(a) Western Blot representative images of acetylated and total H3 histone in cMSCs derived from wt and Egr-1^−/−^ mice when subjected to glucose variations in culture. (b) The densitometry analysis shows that in absence of glucose acetylated protein levels of H3 histone significantly increase in wt but not in Egr-1^−/−^ cMSCs. At increased concentrations of glucose acetylated protein levels of H3 histone significantly decrease compared to the absence of glucose in wt cMSCs, whereas they fairly increased in Egr-1^−/−^ cMSCs. ^#^ *P* < 0.0001.

We show in Figures 6(a) and 6(b) that the protein levels of acetylated H3 histone in wt cMSCs at basal conditions (25 mM) were similar to those of Egr-1^−/−^ cMSCs. Interestingly, in the absence of glucose, the protein levels of acetylated H3 histone were strongly increased in wt but not in Egr-1^−/−^ cMSCs.

The acetylated protein levels of H3 histone were significantly decreased in wt cMSCs in presence of high concentrations of glucose (*P* < 0.001 25 mM and *P* < 0.001 50 mM both* versus* no glucose). whereas only a moderate but not statistically significant increase of the levels could be appreciated in Egr-1^−/−^ cMSCs. In summary, deprivation of glucose in wt cMSCs could be a strong stimulus to induce pan-acetylation of H3 histone compared to high glucose concentrations. Egr-1^−/−^ cMSCs apparently are unable to induce pan-acetylation of H3 histone under a stress condition such as the glucose deprivation. However, further experiments are required to confirm our observations.

## 4. Discussion

Hyperglycemia represents the hallmark sign of diabetes, and it is also an independent risk factor for short- and long-term mortality in acute myocardial infarction. In spite of the recent advances in using MSCs as regenerative therapy for treating cardiac diseases and diabetes, studies providing an exhaustive model based on the “glucose-MSCs-heart triad” are still lacking [21]. Specifically, the improvement of MSC-based therapies for cardiac diseases with diabetes complications must firstly embrace a deeper knowledge on the impact of glucose on MSCs, even prior to their clinical administration. To date, this issue is only partially explored. In fact, understanding the effects of glucose on MSCs does not only represent a main objective to clarify the pathophysiology of hyperglycemia. But, more importantly, it may provide better understanding to enable therapeutic targeting of the impact that glucose has on both the metabolism and the therapeutic potential of the MSC pool.

Physiologically, the cardiac cell metabolism is based on the catabolism of fatty acids, in order to satisfy the contractile function [32]. However, after a myocardial infarction, this scenario is completely overturned. In fact, the myocardium shifts from the basal fatty acid to the glucose metabolism, in order to gain the energy to escape the damage [33, 34]. This adaptive response should include also resident cMSCs.

The effects of glucose on MSCs are still far from being clarified. High glucose has been reported to enhance the proliferation of embryonic stem cells [35] but to decrease that of in rat MSCs [5]. Glucose has no effect on proliferation of human bone marrow-derived MSC, either at short [8] or long exposure durations [3].

More interestingly, high glucose seems not even to be an obstacle for the improvement of MSC-based therapies [36], in spite of the fact that a persistent exposure to elevated levels of glucose can be responsible of the detrimental effects caused by hyperglycemia such as the increased levels of reactive oxygen species and the production of advance glycated end products known as AGEs [37–40]. We confirm that in a murine system cMSC proliferation in low serum is not affected by changing the glucose levels in the medium and show that Egr-1 does not seem to be involved in the proliferative response to glucose. The biological explanation of these observations has likely to be found in a potential “basal glucose tolerance” of this stem cell type. Since glucose tolerance is not mediated by Egr-1, different mechanisms should be involved such as the JAK/STAT or the p38 MAP signaling pathways as described [8].

It is surprising that cMSCs survive even in absence of glucose. Indeed, adipose tissue-derived MSCs have been reported to survive using low ATP levels in presence of severe hypoxia [41]. This is an important biological advantage for MSCs that can be functional in challenging unexpected periods of stress and this in turn would prolong their life in the uncommitted state.

Referring to the impact of glucose on MSC clonogenic ability, it has been shown that high glucose concentration can alter the regenerative potential of MSCs [6] as for the circulating angiogenic cells [42]. We show that increased concentrations of glucose decrease the number of clones generated by wt but not by Egr-1^−/−^ cMSCs, where the role of Egr-1 gene as biological glucose sensor is not preserved.

Given that the combination of low serum and glucose represents a severe metabolic stress for the clonogenic ability, is the lack of Erg-1 functional to preserve the self-renewal properties of MSCs?

In other tissues such as the hematopoietic stem cell (HSC) compartment, Egr-1 is known to be an inhibitor of self-renewal, playing also a crucial role in maintaining HSCs in a quiescent state within the bone marrow [43]. As consequence, Egr-1^−/−^ mice have an increased number of HSCs in the bone marrow [43, 44]. This scenario combined with the glucose context makes possible that the wt cMSC pool protects itself from unnecessary concentrations of glucose by decreasing its clonogenic rate, and thus avoiding the risk of stem cell pool depletion. Egr-1 could participate in this response, acting as both biological glucose sensor and as inhibitor of the self-renewal capacity.

A minimal concentration of glucose must be maintained* in vitro* to guarantee cell survival and viability. Our results show that glucose deprivation is a stronger stimulus to induce Egr-1 upregulation in wt cMSCs compared to other concentrations of glucose. In addition, the widely expressed GLUT-1 and the cardiac specific GLUT-4 glucose transporters mRNA levels are both greatly affected by glucose deprivation, also p300 expression, which has been reported to directly influence both the activity and the function of Egr-1 [45], is affected by glucose deprivation. It is interesting to observe that the phenotype changes of cMSCs in response to glucose variations is completely abolished in Egr-1^−/−^ cases.

To the best of our knowledge, glucose deprivation linked to Egr-1, as reproduced* in vitro* by growing wt and Egr-1^−/−^ cMSCs in cell culture media depleted of glucose, has been not sufficiently examined. Some reports have shown that the deprivation of glucose is able to attenuate neurosphere formation efficiency but no neuronal maturation by human periodontal ligament-derived MSCs [46] and that glucose deprivation-induced necrosis, a distinct hallmark of the metabolic stress present in solid tumors in combination with hypoxia, is specifically regulated by Egr-1 [47]. Moreover, if MSCs are conditioned with glucose depletion, they become even able to enhance their regenerative properties after myocardial infarction [7]. All together our results would indicate that Egr-1 by acting as an early stress response gene functions as a decoder to activate specific responses when glucose is the only substrate available.

Hyperglycemia is known to inhibit the VEGF-VEGF receptor 2 signaling axis, contributing to endothelial dysfunction [48] and causing impairment of the neoangiogenic response in presence of a cardiovascular insult [49]. Our results show that VEGF mRNA levels are upregulated in wt cMSCs conditioned with high glucose concentrations.

This discrepancy could be explained by considering that glucose metabolism is strictly cell type- and tissue dependent and that the glucose mediated effects such as those observed in endothelial progenitor cells [49] may be not reproducible or are differently modulated in other stem cell pools such as MSCs. Specific reports on MSCs indicate that the amount of VEGF released in culture is unmodified by different levels of glucose [8]. More interestingly, the VEGF mRNA levels are not modulated in Egr-1^−/−^ cMSCs. A direct influence of Egr-1 on VEFG has been recently demonstrated in lung cancer cells, either by binding to the proximal region of the VEGF promoter and activating the VEGF expression or by enhancing the hypoxia inducible factor 1alpha- (HIF-1alpha-) mediated VEGF expression [50].

Exposure to high glucose normally increases all three TGF-*β* isoforms in renal cortical fibroblasts [51] as well as in human bone marrow-derived MSCs, thus regulating several cellular processes including proliferation [52]. However, the final effect is cell type dependent. Therefore, TGF-*β*1 can act as inhibitor of cell proliferation in most epithelial cells or as a stimulator of MSC proliferation and expansion [53]. Our data show that increasing the concentration of glucose induces the downregulation of TGF-*β*1 mRNA levels in wt cMSCs. Egr-1 represents a key regulator of TGF*β*-1, which in turn positively regulates the expression of p300 [29]. This would explain why the fluctuation of TGF*β*-1 mRNA levels is suppressed in Egr-1^−/−^ cMSCs and p300 as well.

Several environmental stimuli, including glucose, can ultimately cause epigenetic modifications, which determine gene activation or repression by altering DNA accessibility to transcription factors [11, 54]. In particular, the acetylation state is expression of potential active transcriptional sites. MSCs are not excluded from this process. In adipose tissue-derived MSCs, low levels of acetylated histone H3 and high levels of trimethyl lysine 27 in H3 have been associated with Nkx2.5 and GATA-4 promoters, highlighting a role for the epigenetic modulations in regulating preferential cell lineage commitments [54]. DNA methylation and histone acetylation have been also involved in the control of cartilage differentiation of MSCs [55]. In addition, although in other cell systems such as the vascular endothelial cells, epigenetic modifications have been shown to be caused by transient hyperglycemia, thus providing a plausible explanation for the long lasting effects of diabetes [11]. Specifically, modification of 4 and 9 lysine of the H3 histone tail has recently been linked to the changes in gene expression brought up by hyperglycemia [56].

Egr-1 is known to be involved in epigenetic modifications [57, 58]. However, so far, there are no studies investigating whether glucose variations can induce Egr-1-associated epigenetic changes in cMSCs.

Our data firstly are the first evidence that such epigenetic modifications in cMSCs can occur under glycolytic stimuli and not only in response to hyperglycemia. This observation could be explained by the ability of MSCs, including cMSCs, to modify their phenotype/genotype according to the environmental stimuli and would suggest that Egr-1^−/−^ cMSCs are potentially less “prone” to actively respond to glucose variations.

The histone acetylation is a less stable epigenetic modification than methylation [59]. With the fact that our data are the result of 2 hours of exposure to glucose variations, it can be excluded that they are associated with a “metabolic memory” induced by glucose.

It is clear that the acetylation of H3 histone is not completely abolished in Egr-1^−/−^ mice, and that other mechanisms are involved in the DNA remodeling. However, the downregulation of p300 mRNA levels (a transcriptional coactivator with histone acetyl transferase activity, which has been shown to modify lysine residues on H3 and H4 [11]), together with the low protein levels of acetylated H3 histone found in Egr-1^−/−^ cMSCs, seems to suggest that the mechanism underlying the response to glucose variations in MSCs in relation to Egr-1 is p300 dependent, at least indirectly. We cannot rule out that other partners are involved in such process. For instance, the complex ATF5/p300 has been recently considered important for Egr-1 activation, cell proliferation, and survival [30].

## 5. Conclusions

In conclusion, our data suggest that Egr-1 could play a role as biological glucose sensor in cMSCs in response to glucose variations. Epigenetic changes could represent a key mechanism to determine the glucose-induced response of Egr-1. However, future studies are needed to determine if and which metabolic processes Egr-1 associated are affected in cMSCs.

## Supplementary Material

Figure 2: C-MSC basal growth rate was evaluated by seeding the cells in IMDM/10% FBS in 12-well plates (6.000 cells/cm2). The number of viable cells was obtained by performing a Trypan Blue exclusion assay at 24 and 48 hours.

## Figures and Tables

**Table 1:** 

Target	Sequence
18S forward	AAATCAGTTATGGTTCCTTTGGTC
18S reverse	GCTCTAGAATTACCACAGTTATCCAA
Egr-1 forward	CCTATGAGCACCTGACCACA
Egr-1 reverse	TCGTTTGGCTGGGATAACTC
p300 forward	ACATGATGCCTCGGATGACT
p300 reverse	TAGGGGGCTGTGGCATATT
GLUT-1 forward	CGCAACGAGGAGAACC
GLUT-1 reverse	GCCGTGTTGACGATACC
GLUT-4 forward	GACGGACACTCCATCTGTTG
GLUT-4 reverse	GCCACGATGGAGACATAGC
TGF*β*1 forward	TGGAGCAACATGTGGAACTC
TGF*β*1 reverse	GTCAGCAGCCGGTTACCA
VEGF forward	AAAAACGAAAGCGCAAGAAA
VEGF reverse	TTTCTCCGCTCTGAACAAGG
