# Supplementary figures and images for: Influence of Egr-1 in Cardiac Tissue-Derived Mesenchymal Stem Cells in Response to Glucose Variations
Source: Biomed Res Int. 2014 May 22;2014:254793. doi: 10.1155/2014/254793 (PMC4054710; doi:10.1155/2014/254793)

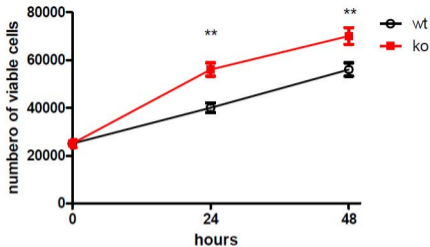

Supplementary figure 2

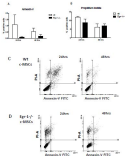

Supplement: Supplementary file 1 — Figure 2: C-MSC basal growth rate was evaluated by seeding the cells in IMDM/10% FBS in 12-well plates (6.000 cells/cm2). The number of viable cells was obtained by performing a Trypan Blue exclusion assay at 24 and 48 hours. [file 254793.f1.pdf]
